# Genetic analysis of local Vietnamese chickens provides evidence of gene flow from wild to domestic populations

**DOI:** 10.1186/1471-2156-10-1

**Published:** 2009-01-08

**Authors:** C Berthouly, G Leroy, T Nhu Van, H Hoang Thanh, B Bed'Hom, B Trong Nguyen, C Vu Chi, F Monicat, M Tixier-Boichard, E Verrier, J-C Maillard, X Rognon

**Affiliations:** 1 CIRAD, UPR AGIRs, Campus International de Baillarguet, 34398 Montpellier Cedex 05, France; 2 AgroParisTech, UMR1236 Génétique et diversité animales, 16 rue Claude Bernard 75321, Paris, Cedex 05, France; 3 INRA, UMR1236 Génétique et diversité animales, 78352 Jouy-en-Josas, France; 4 NIAH, Tu Liem, Ha Noï, Viet Nam

## Abstract

**Background:**

Previous studies suggested that multiple domestication events in South and South-East Asia (Yunnan and surrounding areas) and India have led to the genesis of modern domestic chickens. Ha Giang province is a northern Vietnamese region, where local chickens, such as the H'mong breed, and wild junglefowl coexist. The assumption was made that hybridisation between wild junglefowl and Ha Giang chickens may have occurred and led to the high genetic diversity previously observed. The objectives of this study were i) to clarify the genetic structure of the chicken population within the Ha Giang province and ii) to give evidence of admixture with *G. gallus*. A large survey of the molecular polymorphism for 18 microsatellite markers was conducted on 1082 chickens from 30 communes of the Ha Giang province (HG chickens). This dataset was combined with a previous dataset of Asian breeds, commercial lines and samples of Red junglefowl from Thailand and Vietnam (Ha Noï). Measurements of genetic diversity were estimated both within-population and between populations, and a step-by-step Bayesian approach was performed on the global data set.

**Results:**

The highest value for expected heterozygosity (> 0.60) was found in HG chickens and in the wild junglefowl populations from Thailand. HG chickens exhibited the highest allelic richness (mean A = 2.9). No significant genetic subdivisions of the chicken population within the Ha Giang province were found. As compared to other breeds, HG chickens clustered with wild populations. Furthermore, the neighbornet tree and the Bayesian clustering analysis showed that chickens from 4 communes were closely related to the wild ones and showed an admixture pattern.

**Conclusion:**

In the absence of any population structuring within the province, the H'mong chicken, identified from its black phenotype, shared a common gene pool with other chickens from the Ha Giang population. The large number of alleles shared exclusively between Ha Giang chickens and junglefowl, as well as the results of a Bayesian clustering analysis, suggest that gene flow has been taking place from junglefowl to Ha Giang chickens.

## Background

Molecular tools offer a new approach to investigate both phylogenetic relationships among the sub-species of *Gallus gallus *and the domestication history of the chicken.

According to previous studies of Liu *et al. *[1] and Kanginakudru *et al*. [2], all wild sub-species but one (*G. g. bankiva*) appear closely related. It was concluded that domestication had occurred independently in different locations of Asia, involving *G. g. spadiceus*, *G. g. jabouillei*, and *G. g. murghi*. Furthermore, some genetic exchanges were shown to have occurred between *G. g. murghi *and Indian domestic chickens in recent times (Kanginakudru et al. [2]).

Granevitze *et al. *[3] found a very high genetic diversity in the H'mong chicken breed raised in the northern provinces of Vietnam. The northern province of Ha Giang, at the Chinese border (Yunnan and Guanxi provinces), is part of the distribution area of *G. gallus *[1,4] but it is also considered to be the centre of origin of the H'mong chicken breed. In such a region, forest provides a suitable environment for scavenging chickens, so that local chickens and wild junglefowl coexist, therefore one assumed explanation for the high genetic diversity observed in the H'mong chicken, was possible gene flow from wild populations to domestic chickens.

A Bayesian approach with microsatellite markers has been shown to be useful to provide insight into chicken breed history [5] as well as admixture between sub-species such as taurine and zebu cattle [6,7]. In the present study, we combined microsatellite genotypes from several datasets to address the questions relating to (i) the genetic characteristics of domestic chickens within the Ha Giang province and (ii) possible gene flow between scavenging chickens and wild junglefowl when distribution areas overlap.

## Methods

H'mong chickens can be identified by an extremely black phenotype (involving skin, tarsus and bones). They are raised together with other chickens across the province even if they can be found with higher frequencies in a few communes. In the present study, we carried out a large survey collecting blood samples of 1 082 animals from 30 communes scattered over the Ha Giang (HG) province (22°08' – 23°19' N; 104°33' – 105°33' E). Among the 11 districts, from 2 to 4 communes per district (30 in total) and 3 to 8 villages per commune (190 in total) were surveyed. Sampling included chickens showing either the H'mong phenotype or any other phenotype that were raised together in backyards.

Genomic DNA was extracted from blood using the QIAamp Kit from QIAGEN. The PCR products were labelled with the fluorescent dyes and genotyped using a capillary sequencer (Beckman Coulter CEQ8000). Among the 30 microsatellites recommended by FAO/ISAG and available for genotyping in the NIAH laboratory (Ha Noï), reliable genotypes were obtained with 20 markers for HG chickens (data available upon request).

This data set was combined with two other ones: (i) a subset already studied by Berthouly *et al. *[8] involving 2 wild populations of *G. gallus *(captured in northern Thailand in 1997, reared in a field station of the University of Chiang Mai and sampled in 1999), 6 local standardised Asian breeds and 2 commercial lines; (ii) a second set with 1 population of F2 animals from *G. gallus *(captured in Vietnam in 1997 and conserved in a French zoological park), and 3 other commercial lines (Table 1). Among commercial lines, the white-egg layers correspond to the White Leghorn breed, an ancient Mediterranean type of breed, whereas brown-egg layers and broilers have Asian origins following importation from Asia to Europe and the USA in the 19^th ^century. Sampling of wild junglefowl from the Ha Giang province was not possible for technical reasons.

**Table 1:** Summary of genetic diversity measures across wild and domestic populations

Code	Breed name	Sample Size	H_Exp_	H_Obs_	A	F_IS_
BS_LD	Broiler sire Line D	30	0.46	0.45	2.5	0.01
BS_LC	Broiler Sire Line C	25	0.47	0.49	2.2	-0.03
BD_LB	Broiler dam Line B	25	0.47	0.47	1.6	0.00
BE_LC	Brown-egg Layer C	25	0.41	0.36	2.2	0.11
WE_LA	White-egg Layer A	25	0.27	0.27	2.1	-0.02
Gg1	*G. g. spadiceus. *Thailand*	16	0.60	0.58	2.2	0.05
Gg2	*G. g.gallus. *Thailand*	15	0.62	0.66	2.4	-0.06
Gg3	*G. g. gallus. *Vietnam	6	0.57	0.70	2.7	-0.27
HT	Hua-Tung. Taiwan	45	0.55	0.57	2.8	-0.02
JC	Ju-Chi. Taiwan	48	0.40	0.42	2.0	-0.05
KM	Quemoy. Taiwan	47	0.49	0.47	2.3	0.04
HY	Hsin-Yi. Taiwan	47	0.50	0.51	2.1	-0.02
SK	Shek-Ki. China	46	0.44	0.40	2.0	0.10
NG	Nagoya. Japan	48	0.42	0.43	2.2	-0.02

**HG**	**Ha Giang. Vietnam **(mean values)		**0.62**	**0.55**	**2.9**	**0.12**

Ha Giang (HG) detailed for the 30 communes						

HG1	Lung-Pu	36	0.63	0.49	2.9	0.22
HG2	Ma-Le	9	0.61	0.54	2.8	0.13
HG4	Lung-Cu	44	0.62	0.52	2.9	0.17
HG7	Thai Phin Tung	6	0.70	0.62	3.3	0.12
HG16	Pho-Cao	39	0.57	0.52	2.7	0.08
HG20	Bach-Dich	23	0.65	0.59	3.0	0.08
HG25	Na-Khe	22	0.57	0.49	2.7	0.15
HG40	Tat-Nga	26	0.69	0.57	3.2	0.18
HG48	Khau-Vai	47	0.61	0.53	2.6	0.13
HG49	Tung-Vai	52	0.61	0.55	2.8	0.09
HG56	Quyet-Tien	24	0.58	0.52	2.8	0.11
HG61	Lung-Ho	37	0.58	0.54	2.7	0.08
HG65	Du-Gia	9	0.68	0.59	2.7	0.14
HG72	Ngoc-Duong	16	0.64	0.59	3.1	0.07
HG75	Giap-Trung	52	0.61	0.53	2.9	0.14
HG85	Po-Lo	90	0.63	0.55	2.8	0.13
HG88	Minh-Ngoc	32	0.64	0.57	2.9	0.11
HG91	Nan-Xin	64	0.61	0.56	3.0	0.08
HG95	Phu-Linh	32	0.61	0.51	2.8	0.17
HG103	Chi-Ca	88	0.65	0.58	2.8	0.10
HG108	Thuong-Son	16	0.62	0.53	3.0	0.14
HG110	Po Ly Ngai	49	0.61	0.56	2.9	0.09
HG113	Yen-Cuong	52	0.63	0.58	2.8	0.06
HG114	San Sa Ho	44	0.62	0.58	2.9	0.06
HG145	Quang-Nguyen	25	0.62	0.55	2.8	0.10
HG146	Trung Thanh	14	0.61	0.54	2.8	0.13
HG157	Tan-Nam	14	0.65	0.57	2.8	0.12
HG169	Quang-Minh	15	0.62	0.54	3.0	0.15
HG179	Xuan-Giang	88	0.64	0.55	2.9	0.14
HG184	Vinh-Phuc	17	0.60	0.51	2.9	0.15

A: allelic richness; * pooled as one population in [8]

These two subsets were genotyped by the LABOGENA laboratory (France). In order to calibrate allele sizes between the two laboratories, a set of 17 reference animals within the 14 external populations was analysed jointly with the animals from the Ha Giang province. The difference in allele size observed between laboratories was adjusted according to Berthouly *et al. *[8]. Eighteen markers, for which allele sizes were consistent from one laboratory to another, were used for genetic analysis (see Additional file 1).

Allele frequencies and expected and observed heterozygosity were calculated using GENETIX [9]. Allelic richness by rarefaction was estimated using FSTAT [10]. GENEPOP [11] was used to compute *F-statistics *[12] and departure from Hardy-Weinberg equilibrium using exact tests. Test significance was corrected with sequential Bonferroni correction on loci. The matrix of F_ST _Latter's distance [13] between breeds was calculated to draw a NEIGHBORNET tree using SPLITSTREE 4.8 [14].

We investigated the genetic structure of the sampled populations using a Bayesian clustering procedure implemented in STRUCTURE [15], with the admixture method and correlated allele frequency version of the programme [16]. First, we performed our analysis only using the HG sample. We did 15 runs for each different value of *K *with 10^5 ^iterations following a burn-in period of 300 000 assuming that the data set could be represented by *K *separate genetic clusters (*K *= 1 to 15).

Second, we analysed the clustering of HG chickens with the other fourteen breeds. In order to avoid bias due to sample size, we reduced the HG sample to 32 randomly selected animals with at least 1 animal per commune, before applying the procedure of Rosenberg *et al. *[5] using 50 runs (60 000 iterations; burn-in period of 40 000). The Q-matrix of the run with the highest similarity over all runs using the similarity function G' was computed for each *K *using CLUMPP [17].

The two analyses above were done to estimate the number of genetic clusters (*K*) within the Ha Giang province and within the global dataset. Thus, values of *K *were assessed according to Evanno *et al. *[18].

Afterwards a third analysis was conducted to highlight admixture pattern. The same approach as the second analysis was done but with all HG chickens using 50 runs (60 000 iterations; burn-in period of 40 000) from *K *= 2 to *K *= 15. The Q-matrix of the run with the highest similarity was also computed.

Admixture rate between the four communes and wild populations was estimated using LEADMIX [19]. It performs maximum likelihood estimation of admixture proportions in a model where the ancestral species *P*_0 _is split into two parental populations *P*_1 _(wild *G. gallus*) and *P*_2 _(HG chickens from non admixed communes) that evolved independently before they contributed in genetic proportion *p*_1 _and (1-*p*_1_) to form a hybrid population *P*_*h *_(the four Ha Giang's admixed communes).

## Results and discussion

### Genetic characterisation of the chicken population from the Ha Giang province

The highest value for expected heterozygosity (> 0.60) was found in HG chickens and in the wild junglefowl populations from Thailand (Table 1). The observed heterozygosity was the highest for wild populations, averaging 0.64. For HG chickens, *H*_*o *_ranged from 0.51 to 0.62 according to the sampled communes, and was similar to values previously observed for other local populations [20-22]. HG chickens exhibited the highest allelic richness (mean A = 2.9) and harboured 33 of the 36 private alleles found across all populations. Moreover, 13 other alleles were shared only by the wild and HG populations.

Within the Ha Giang province, *F*_*IS *_averaged 0.121, with 0 to 4 loci showing heterozygote deficiency. Among the 30 communes, only two did not exhibit a significant deviation from HWE (Table 1). The remaining Asian breeds and commercial lines reached mean *F*_*IS *_values of 0.004 and 0.15 respectively (Table 1).

Multilocus between breed diversity accounted for 12.9% of total diversity. Within the Asian group (i.e. 6 Asian breeds and HG chickens), genetic differentiation between breeds averaged 24.3% whereas within commercial lines it reached 36.9%. HG chickens had the lowest mean *F*_*ST *_value with the wild populations (ranging from 0.117 to 0.172).

Within the Ha Giang province, only 3.7% of the genetic diversity was due to differentiation between communes. The best likelihood was found for *K *= 4 according to Evanno *et al. *[18] using the Bayesian approach (see Additional file 2). However maximum mean *q *values per commune ranged from 0.352 (HG146) to 0.948 (HG65). We found that within a given commune, animals belong to 2 to 4 clusters except for the commune HG65 for which all animals belong to one population. Thus no reliable genetic subdivision was observed after performing the Bayesian approach. Since villages are distant and separated from each other by forest or wide land crop areas, village poultry stocks within a commune may behave as a small genetic unit, which is in agreement with the high *F*_*IS *_values observed. However, commercial exchanges often take place for poultry replacement after epidemic events, explaining the low *F*_*ST *_values and results obtained with the Bayesian approach. Q-matrix did not show any specific genetic clustering according to the individual phenotypes (i.e H'mong and non H'mong). Therefore, both phenotypes may be considered as part of a single population, as observed for other local chicken populations in Africa [21,22]. This was consistent with the fact that the determinism of black skin and bones involves only two major genes, *FM *for fibromelanosis (an autosomal dominant mutation) and *ID *for inhibition of dermal melanin (sex-linked with a recessive wild-type allele for grey shank) [23]. Thus the segregation of mutations at these two loci may easily explain that black skin chickens are distributed all over the population.

### Clustering and admixture approach

**Figure 1:**
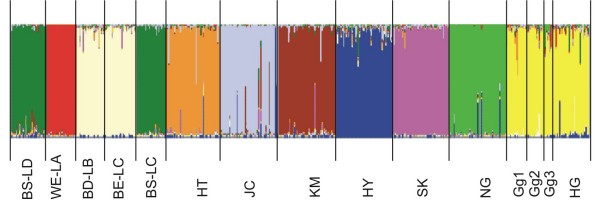
**Clustering diagrams of the 14 chicken populations and the reduced sample of the Ha Giang chickens obtained for *K *= 10**. Each individual is represented by a vertical line, which is partitioned into *K *= 10 colored segments that represent the individual's estimated membership fractions in *K *clusters using the Q matrix of the run with the best similarity. Black lines separate individuals of different populations coded as defined in Table 1.

When using the reduced HG sample, the log likelihood value reached a plateau at *K *= 10 (see Additional file 3A). For further *K *values, higher Δ*K *were observed indicating instability across runs [Pritchard]. Following Evanno *et al. *[18], the highest values were obtained for *K *= 2, *K *= 3 and *K *= 10 (see Additional file 3B). Leroy *et al. *[24] hypothesised that the highest values obtained for small *K *are biased with Evanno's method when the number of breeds was important in the dataset. Therefore, using both approaches, the highest likelihood was obtained for *K *= 10 (Fig. 1). The two broiler lines (BS-LD and BS-LC) could not be distinguished. The BD-LB, a broiler dam line, clustered with the BE-LC layer line, which came from the same commercial breeder. All 6 Asian breeds were well separated from each other as previously observed in Berthouly et al. [8]. Ha Giang chickens and the wild populations segregated for most of the runs (data not shown) in the same cluster. Considering the wild samples, it could be assumed that these populations may have been subjected to important founder effects, but were not very much affected by genetic drift because of their recent introduction into experimental farms in Thailand or in a zoological park in France. However, the three populations exhibited the same admixture pattern and constituted a genetically homogeneous group. Thus, these wild samples from different geographic origins (i.e. Thailand and Vietnam), could be considered as a good representation of the genetic diversity of *G. gallus *in South-East Asia. The population of HG chickens was the only Asian population that clustered with *G. gallus*. The same number of chickens was considered for HG chickens as well as for the other breeds, therefore the result was not biased by differences in sample size. Although Asian breeds were under conservation and might have been subjected to a founder effect, they all had South-East Asian origin and were still showing a high genetic diversity, as in the HT breed. Therefore, the clustering pattern clearly shows a genetic proximity of HG chickens with wild red jungle fowl.

**Figure 2:**
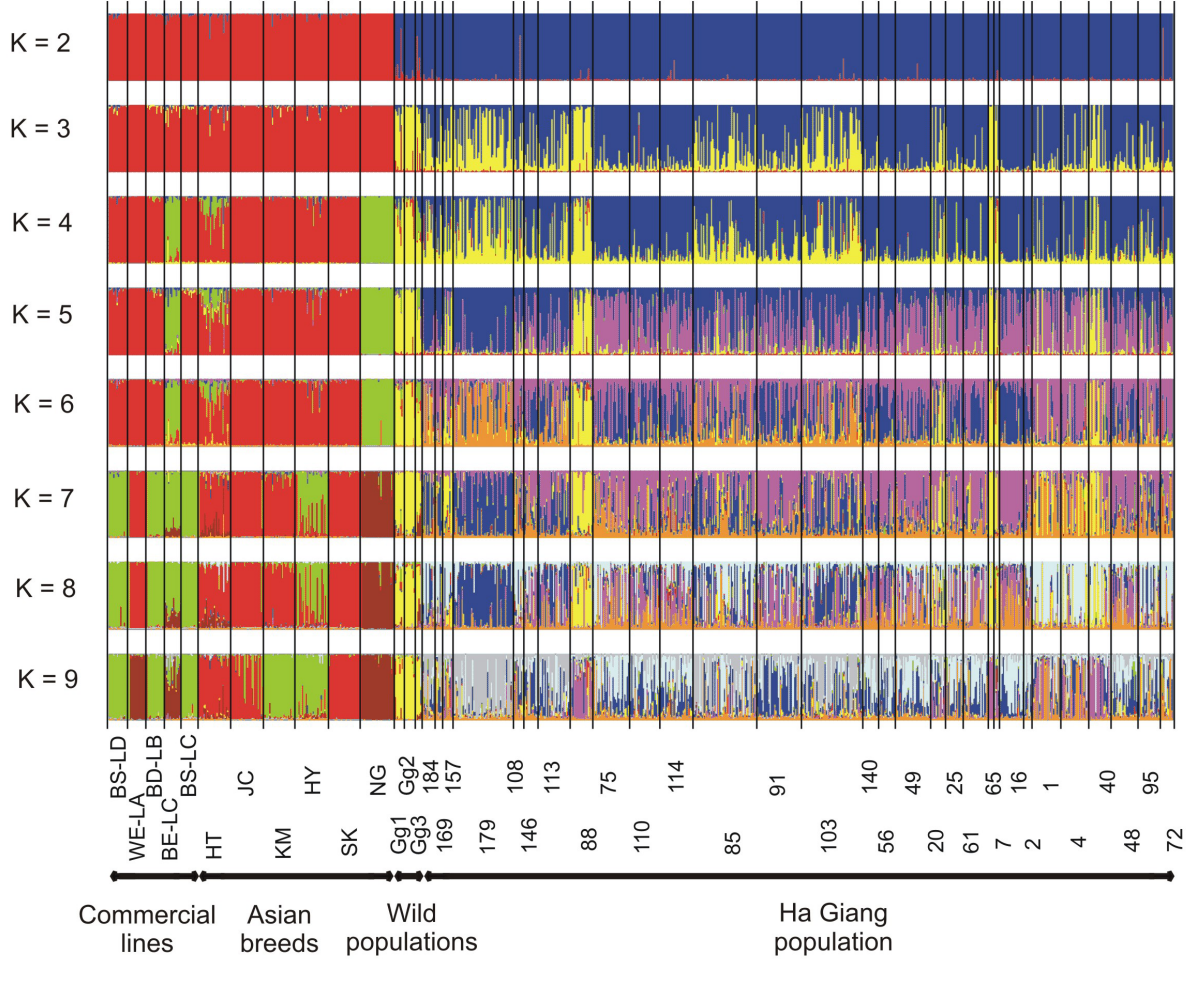
**Clustering diagrams of 14 chicken populations and the entire sample of the Ha Giang chickens obtained from *K *= 2 to *K *= 9 using Q matrices of runs with best similarities**.

In order to focus on this genetic proximity, all the samples from the 30 communes were added to the analysis (Fig. 2). Similarity coefficients computed over 50 runs were high and ranged from 0.70 (*K *= 8) to 0.99 (*K *= 2). Following Rosenberg *et al. *[5], we focused on the analysis of the clustering order and admixture pattern, from K = 2 to 9. For *K *= 2, cluster 1 grouped the commercial lines and the 6 Asian breeds, the HG chickens formed cluster 2 and the three wild populations admixed with both clusters. Starting from *K *= 3, the two previous clusters remained and the new one (in yellow on Fig. 2) represented part of the wild populations. For *K *= 4, the Japanese NG and BE-LC breed separated from the other breeds until K = 7 for which the NG started to be clearly identified. For *K *= 7, structuring between Asian breeds and commercial lines appeared and admixture of two Asian and the broiler lines was found at *K *= 9. No Vietnamese communes admixed with Asian breeds nor with commercial lines. Thus, the HG population seemed to be a local population, which had not been submitted to any recent introgression of exotic or other Asian breeds. Starting from *K *= 4, undistinguished clusters appeared for HG chickens but four communes (HG88, HG65, HG7, HG40) always shared the same admixture pattern with the three sets of wild junglefowl (in yellow). For *K *= 9, animals from the four communes that clustered with wild populations at lower K values, clustered together in a new cluster.

**Figure 3:**
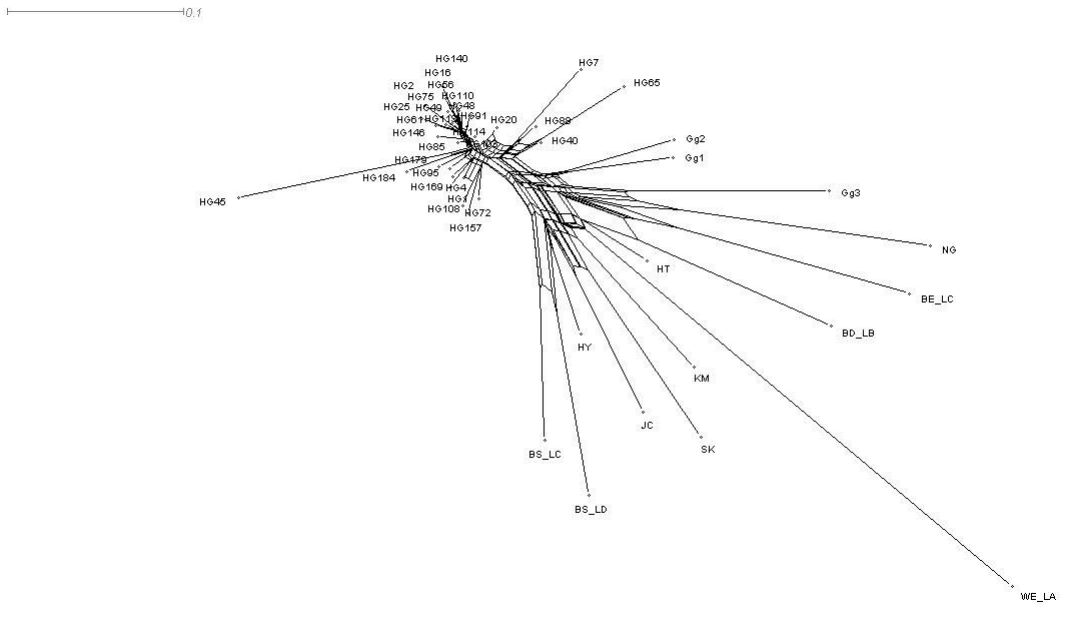
**Neighbornet tree for the 44 populations using the Latter's genetic distance**.

Furthermore chickens from these communes were found isolated from the other ones and closely related to the wild populations, when drawing a neighbornet tree with Latter's genetic distance (Fig. 3). Since no similar admixture pattern was observed in the remaining communes, such a pattern could be considered as a signature of local gene flow from wild to domestic chickens. The mean q probability of animals from these four communes to belong to the wild cluster ranged from 0.75 to 0.89 for *K *= 8.

Admixture rate from LEADMIX was estimated with our sample of wild populations used as the introgressive population. The admixture rate reached 0.625, with a 95% CI ranging between 0.424–0.986, indicating that some of these chickens would be more related to wild chickens than to domestic chickens. This rate may be biased due to the violation of a few assumptions. The first one is that the model implemented in LEADMIX does not assume a constant migration but a single admixture event. In the present situation, where both populations are coexisting, a constant migration from wild to domestic chickens is most probable. This would also affect the minimum value of genetic drift allowed by the programme. If admixture occurred recently, genetic drift could be negligible. Secondly, we assumed that our wild sample was the true population introgressing the domestic chicken. However this wild parent is obviously not the true one, since it did not originate from the Ha Giang province and, also it may have been subjected to a strong founder effect. A second analysis, using an unknown wild parental population, led to a similar result with an admixture rate reaching 0.75, with a 95% CI ranging between 0.652–0.807. The explanations for these high admixture rates, taking place in these four communes close to the forest, are (i) free scavenging chickens can easily be reproduce with wild ones and (ii) a few householders unofficially explained during their interviews that they used to pick up eggs in the forest and raised the chicks. Gene flow from wild to domestic chickens occurred in a significant way in the province and may constitute one of the reasons for the observed high genetic diversity. Gene flow in Indian flocks, raised in similar conditions (i.e. scavenging and forest), has been previously reported by Kanginakudru *et al. *[2] but it was assumed to be low. However, it might be underestimated because of the limited number of samples. The large scale sampling, done within an area where both domestic and wild chickens co-existed, allowed us to reveal more precisely the extent of this gene flow, which concerned 6.7% of the sampled chickens.

Important commercial exchanges of chickens within the province led to some homogenization of the gene pool, which is in accordance with the low *F*_*ST *_values between communes, and with the absence of any genetic substructure found with the Bayesian approach. Also, frequent exchanges will allow the spreading of alleles of wild origin across the province. This would explain the absence of specific private alleles shared with wild populations in these four specific communes, as would be expected. However, gene flow would increase gene diversity, which is in accordance with the highest values of *H*_*e *_observed in 3 of the four communes.

## Conclusion

The Ha Giang chicken population shows high genetic diversity which is due in part to the farmer practices (i.e. commercial exchanges, low selection). This genetic diversity is also increased by gene flow occurring from wild to domestic chickens. This could also have occurred in another way and lead to a genetic endangerment of Red Jungle fowl. Furthermore, providing evidence of gene flow is also of prime interest for studies on the risk of disease diffusion between wild and domestic populations.

## Authors' contributions

NVT and HTH carried out sample collection; BTN did the DNA extraction, PCR and sequencing; BB participated in the laboratory protocols and manuscript revision; CB carried out sample collection, sequencing, the computational analysis and prepared the manuscript; GL participated in the computational analysis; MTB contributed to the description of the populations from the AvianDiv consortium and the revision of the manuscript; XR participated in the computational analysis and preparation of the manuscript; FM participated in the conception of the study; EV participated in the design of the study and the revision of the manuscript; VCC participated in the coordination of the study; JCM participated in the design, coordination of the study, and revision of the manuscript.

## Supplementary Material

Additional file 1 **Summary of polymorphic measures for microsatellite markers**. For each marker, the following information is given: allele range, number of alleles, number of private alleles, expected and observed heterozygosity, number of populations with heterozygote deficiency and heterozygote excess and *F-statistics*. Click here for file

Additional file 2 **Evolution of ΔK. the ΔK is calculated as ΔK = m|L"(K)|/s [L(K)] for the 30 commune populations of the Ha Giang province**. Click here for file

Additional file 3 **Evolution of Log likelihood and ΔK. A): Log likelihood evolution across *K *values for the 15 populations (i.e. reduced sample of HG chickens)**. B) ΔK calculated as ΔK = m|L"(K)|/s [L(K)] for the 15 populations (i.e. reduced sample of HG chickens) ΔK calculated as ΔK = m|L"(K)|/s [L(K)] for the 15 populations (i.e. reduced sample of HG chickens). Click here for file
