# Supplementary material for: Genetic analysis of local Vietnamese chickens provides evidence of gene flow from wild to domestic populations
Source: BMC Genet. 2009 Jan 8;10:1. doi: 10.1186/1471-2156-10-1 (PMC2628941; doi:10.1186/1471-2156-10-1)
Supplement: Additional file 1 — Summary of polymorphic measures for microsatellite markers. For each marker, the following information is given: allele range, number of alleles, number of private alleles, expected and observed heterozygosity, number of populations with heterozygote deficiency and heterozygote excess and F-statistics. [file 1471-2156-10-1-S1.doc]

Additional file 1: Summary of polymorphic measures for microsatellite markers.

Description: For each marker, the following information is given: allele range, number of alleles, number of private alleles, expected and observed heterozygosity, number of populations with heterozygote deficiency and heterozygote excess and *F-statistics*

| Locus | Range | A | Ap | H_Exp_ | H_Obs_ | D_HWE_ | E_HWE_ | F_IS_ | F_ST_ | F_IT_ |
| --- | --- | --- | --- | --- | --- | --- | --- | --- | --- | --- |
| ADL112 | 120-130 | 6 | 0 | 0.528±0.148 | 0.500±0.151 | 4 | 0 | 0.079 | 0.120 | 0.189 |
| ADL268 | 100-114 | 6 | 0 | 0.629±0.100 | 0.628±0.102 | 0 | 0 | 0.014 | 0.190 | 0.201 |
| ADL278 | 111-127 | 8 | 4 (2xHG,Gg3,Gg1) | 0.560±0.123 | 0.507±0.105 | 2 | 0 | 0.103 | 0.196 | 0.279 |
| MCW034 | 214-248 | 16 | 4 (HG) | 0.706±0.153 | 0.670±0.150 | 4 | 0 | 0.062 | 0.164 | 0.216 |
| MCW037 | 151-157 | 4 | 0 | 0.622±0.130 | 0.541±0.130 | 3 | 0 | 0.139 | 0.106 | 0.230 |
| MCW067 | 169-179 | 6 | 1(Gg3) | 0.638±0.147 | 0.601±0.150 | 1 | 0 | 0.067 | 0.100 | 0.160 |
| MCW069 | 155-173 | 10 | 1(HG) | 0.719±0.171 | 0.679±0.173 | 0 | 1 | 0.040 | 0.091 | 0.128 |
| MCW078 | 134-144 | 6 | 2(HG) | 0.433±0.183 | 0.360±0.178 | 10 | 0 | 0.229 | 0.147 | 0.342 |
| MCW081 | 110-134 | 8 | 2(HG) | 0.455±0.165 | 0.440±0.159 | 2 | 0 | 0.068 | 0.217 | 0.271 |
| MCW098 | 252-258 | 4 | 2(HG) | 0.275±0.125 | 0.250±0.120 | 0 | 0 | 0.037 | 0.087 | 0.121 |
| MCW111 | 94-114 | 11 | 3(HG) | 0.681±0.143 | 0.665±0.136 | 3 | 0 | 0.053 | 0.091 | 0.140 |
| MCW183 | 293-333 | 18 | 8(HG) | 0.550±0.165 | 0.526±0.164 | 0 | 0 | 0.070 | 0.107 | 0.170 |
| MCW216 | 136-148 | 7 | 2(HG) | 0.482±0.180 | 0.363±0.167 | 4 | 0 | 0.240 | 0.157 | 0.360 |
| MCW222 | 216-224 | 5 | 1(HG) | 0.468±0.153 | 0.375±0.140 | 4 | 0 | 0.187 | 0.135 | 0.297 |
| MCW248 | 213-223 | 6 | 2(HG) | 0.640±0.166 | 0.594±0.166 | 0 | 0 | 0.074 | 0.098 | 0.165 |
| MCW295 | 84-106 | 12 | 2(HG) | 0.712±0.129 | 0.631±0.122 | 2 | 0 | 0.102 | 0.100 | 0.192 |
| MCW330 | 256-288 | 9 | 2(HG) | 0.689±0.195 | 0.638±0.198 | 2 | 0 | 0.085 | 0.118 | 0.192 |
| LEI166 | 344-354 | 6 | 3(HG) | 0.562±0.134 | 0.539±0.134 | 1 | 0 | 0.042 | 0.094 | 0.131 |
| Global |  | 148 | 36 |  |  | 42 | 1 | 0.090 | 0.129 | 0.208 |

A: Number of alleles; Ap: number of private alleles (Breed); D_HWE_: number of populations with heterozygote deficiency; E_HWE_: number of populations in Heterozygotes excess; HG: Ha Giang chicken population
