# Supplementary material for: Genetic analysis of local Vietnamese chickens provides evidence of gene flow from wild to domestic populations
Source: BMC Genet. 2009 Jan 8;10:1. doi: 10.1186/1471-2156-10-1 (PMC2628941; doi:10.1186/1471-2156-10-1)

Additional file 2: Evolution of ΔK

Description: the ΔK is calculated as ΔK = m|L′′(K)|/ s[L(K)] for the 30 commune populations of the Ha Giang province


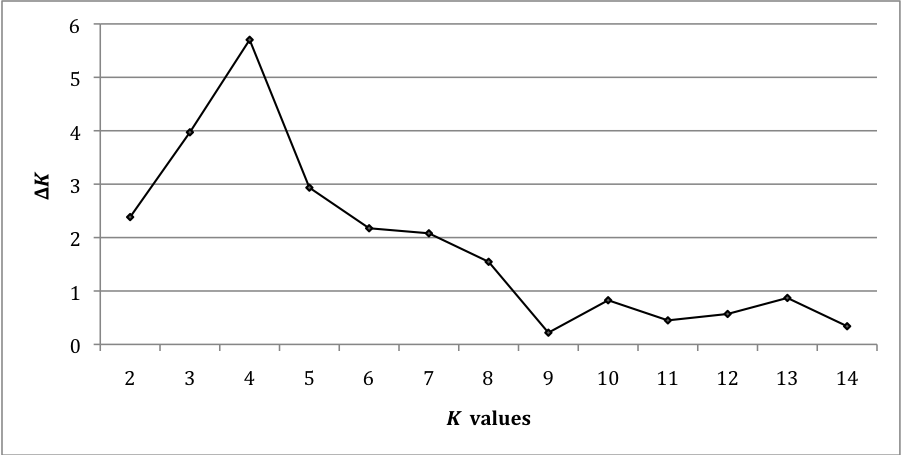

Supplement: Additional file 2 — Evolution of ΔK. the ΔK is calculated as ΔK = m|L"(K)|/s [L(K)] for the 30 commune populations of the Ha Giang province. [file 1471-2156-10-1-S2.doc]
