# Supplementary material for: Genetic analysis of local Vietnamese chickens provides evidence of gene flow from wild to domestic populations
Source: BMC Genet. 2009 Jan 8;10:1. doi: 10.1186/1471-2156-10-1 (PMC2628941; doi:10.1186/1471-2156-10-1)

Additional file 3: Evolution of Log likelihood and ΔK

Description: A): Log likelihood evolution across *K* values for the 15 populations (i.e. reduced sample of HG chickens).B)ΔK calculated as ΔK = m|L′′(K)|/ s[L(K)] for the 15 populations (i.e. reduced sample of HG chickens)ΔK calculated as ΔK = m|L′′(K)|/ s[L(K)] for the 15 populations (i.e. reduced sample of HG chickens)

A


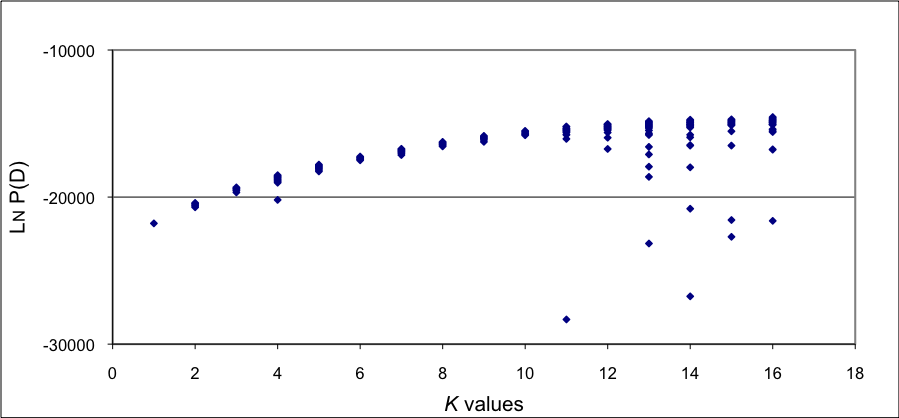


B


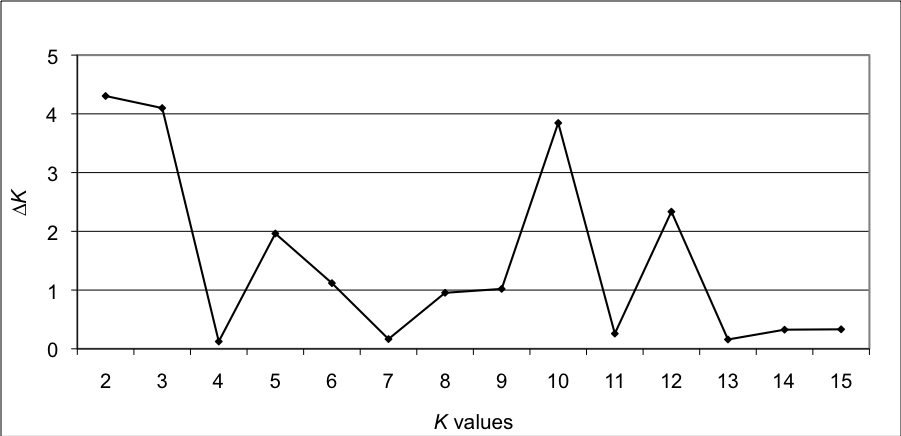

Supplement: Additional file 3 — Evolution of Log likelihood and ΔK. A): Log likelihood evolution across K values for the 15 populations (i.e. reduced sample of HG chickens). B) ΔK calculated as ΔK = m|L"(K)|/s [L(K)] for the 15 populations (i.e. reduced sample of HG chickens) ΔK calculated as ΔK = m|L"(K)|/s [L(K)] for the 15 populations (i.e. reduced sample of HG chickens). [file 1471-2156-10-1-S3.doc]
